# p70S6K is regulated by focal adhesion kinase and is required for Src-selective autophagy

**DOI:** 10.1016/j.cellsig.2015.05.017

**Published:** 2015-09

**Authors:** Emma Sandilands, Christina Schoenherr, Margaret C. Frame

**Affiliations:** Edinburgh Cancer Research UK Centre, Institute of Genetics and Molecular Medicine, University of Edinburgh, Western General Hospital, Crewe Road South, Edinburgh EH4 2XR, United Kingdom

**Keywords:** FAK, PDK1, Akt, p70S6K, S6, Autophagosomes

## Abstract

Here we report that focal adhesion kinase (FAK) is required for optimal signalling to the Akt-p70S6K-S6 pathway in squamous cell carcinoma (SCC) cells. Specifically, in SCCs that are genetically deficient for FAK, there is reduced phosphorylation of Akt, p70S6K and S6, and signalling to Akt-p70S6K-S6 is more sensitive to inhibition by multiple agents that suppress the pathway. By contrast, mTOR is unaffected. Indeed, pharmacological agents that inhibit the Akt-p70S6K-S6 pathway, and PDK1 that lies upstream of Akt, also impair the autophagic targeting of activated c-Src (p-Src) in FAK deficient cells. This is associated with loss of a complex between p-Src and the autophagy protein LC3, a biochemical surrogate of impaired Src-selective autophagy. In keeping with a vital role for p70S6K, inhibition by a selective inhibitor and specific siRNA also impaired Src-selective autophagy. Finally, components of the PDK1-Akt-p70S6K signalling pathway were co-located with p-Src at autophagosomes, and Src and p70S6K co-exist in the same biochemical complex. We therefore deduce that the FAK-regulated signalling module PDK1-Akt-p70S6K that controls Src's intracellular trafficking operates at Src-containing autophagosomes.

## Highlights

• Signalling to p70S6K is FAK dependent in squamous carcinoma cells.
• FAK deficient cells are more sensitive to inhibition of the Akt-p70S6K-S6 pathway.
• PDK1-Akt-p70S6K signalling controls Src trafficking to autophagosomes.
• Src selective autophagy requires p70S6K but not mTOR.
• PDK1, Akt and p70S6K co-localise with active-Src autophagosomes.

## 1 Introduction

Src is a member of a family of non-receptor tyrosine kinases that control cell adhesion, invasion, proliferation, survival and angiogenesis [1]. Overexpression or activation of Src proteins occurs in a number of human cancers and is frequently linked to poor clinical outcome [2–4], although the molecular mechanisms that cancer cells use to maintain and cope with elevated levels of promiscuous activated tyrosine kinases like Src are poorly understood.

Src consists of a myristoylation domain, unique and Src homology 3 and 2 domains (SH3 and SH2), a kinase domain containing an autophosphorylation site at Tyr 416 (Y416) and a C-terminal regulatory region. Activation results in phosphorylation of Y416 and allows the SH3 and SH2 domains to bind to important partner proteins, including focal adhesion kinase (FAK). Recently we perturbed flux through the Src/FAK pathway in SCC cells, and identified a new mechanism for regulating high levels of active Src kinase [5]. Briefly, genetic deletion of the gene encoding FAK, detachment of FAK-proficient cells or expression of non-phosphorylatable FAK proteins promotes trafficking of active Src from peripheral focal adhesions to cytoplasmic puncta that contain autophagy proteins. Sequestration in these untypically regulated autophagosomes requires the formation of a complex between Src and LC3B, a known autophagy cargo receptor [6,7], and this is mediated by c-Cbl [5]. Disruption of this process results in restoration of active Src at peripheral adhesions and loss of the LC3B/Src complex. This autophagic targeting of Src allows these FAK deficient cancer cells to deal with elevated levels of active Src kinase that is not spatially controlled by its tethering partner FAK [5].

Typically, engagement of autophagy occurs during metabolic stress when cells need to reallocate nutrients, or when specific protein aggregates, mis-folded proteins, organelles, bacteria or viruses need to be removed. This process involves a number of distinct steps that are all tightly regulated by a family of autophagy-related (ATG) proteins, the secretory and endocytic pathways, the cytoskeletal network, transcription factors, post-translational modifications and protein kinase signalling cascades [8,9]. What we described was an adhesion stress-induced form of autophagy that does not seem to be dependent upon nutrient starvation [5] and was highly selective for the targeting of FAK-tethered oncogenic kinases, such as Src and Ret [5,10]. In the current work, we identified the signalling requirements of Src-selective autophagy. We found that FAK regulates phosphorylation of Akt, p70S6K and S6, and that components of the PDK1-Akt-p70S6K-S6 signalling pathway were co-located in Src-containing autophagosomes. Crucially, the activity of p70S6K, but not mTOR, was required for Src-selective autophagy triggered by FAK loss.

## 2 Materials and methods

### 2.1 Antibodies

Antibodies used were: anti-p-Src Y416, anti-Src 36D10, anti-LC3B, anti-FAK, anti-p-PDK1 S241, anti-PDK1, anti-p-Akt S473, anti-Akt, anti-GAPDH, anti-p-mTOR S2448, anti-p-mTOR S2481, anti-mTOR, anti-p-S6 S235/236, anti-p-S6 S240/244, anti-S6, anti-p-p70S6K T389, anti-p70S6K, anti-mouse and anti-rabbit IgG-peroxidase conjugated secondary antibodies (all New England Biolabs, Herts, UK), anti-LC3 (MBL, MA, USA), and anti-paxillin (BD Transduction Laboratories, Oxford, UK). Alexa 488 and Alexa 594 conjugated secondary antibodies, Deep Red Cell Mask and Vectashield microscope mounting medium with DAPI were from Invitrogen (Paisley, UK).

Tocriscreen Kinase Inhibitor Toolbox, SB218078, SB216763, ZM306416, PD407824, BIO, Ki8751 and PF4708671 were all from R & D (Abington, UK), hygromycin B from Merck Biosciences (Nottingham, UK), dasatinib from Bristol Myers Squibb (Middlesex, UK) and 3MA from Sigma (Poole, UK). The Micro BCA Protein Assay Kit was from Pierce Ltd. (Northumbria, UK), Lipofectamine2000 from Invitrogen (Paisley, UK) and HiPerFect from Qiagen (Crawley, UK). siRNA was from Dharmacon (Colorado, USA).

### 2.2 Generation of FAK-deficient squamous cell carcinoma cells

K14*Cre*ER^T2^/FAK*^flox/flox^* mice were subjected to chemical carcinogenesis [11] and FAK positive cells grown from the resultant tumours as described previously [5]. FAK was deleted using 10 μM of 4-hydroxy tamoxifen (4-OHT) and stable single cell clones generated that were FAK deficient (FAK −/−) [5].

### 2.3 Cell culture and transfection

Cells were cultured in Glasgow Minimum Essential Medium (10% foetal calf serum, 2 mM l-glutamine, NEAA, sodium pyruvate and MEM vitamins). Phoenix Eco cells were transfected with pWZL-FAK-WT using Lipofectamine2000 (as per manufacturers instructions) and the supernatant from these cells used to infect FAK −/− SCC using 10 μg/ml polybrene. These FAK-WT SCC cells were then selected and maintained in 1 mg/ml hygromycin B.

### 2.4 Kinase inhibitor screen

FAK −/− and FAK-WT SCC cells were plated on flat-bottomed 96-well glass plates overnight. Then 80 kinase inhibitors from the Tocriscreen Kinase Inhibitor Toolbox were added at 1 μM, 10 μM or 20 μM for 24 h. Plates were washed in TBS and fixed for 10 min (formaldehyde (3.7%), K-Pipes pH 6.8 (100 mM), EGTA (10 mM), MgCl_2_ (1 mM), Triton X-100 (0.2%)). After three washes (in TBS containing 0.1% Triton X-100) plates were blocked in TBS containing 0.1% BSA for 1 h prior to overnight incubation with anti-p-Src Y416. Primary antibody was washed off and a fluorescently labelled secondary antibody (Alexa 488) and Deep Red Cell Mask were added for 45 min. After three washes (TBS with 0.1% Triton X-100) DAPI (diluted 1 in 1000 in PBS) was added for 10 min. Plates were washed twice in PBS and each well imaged using an Olympus Scan-R microscope.

Data were analysed using Scan-R analysis software to calculate the number of puncta (based on size and shape of Src positive puncta) per cell (calculated using DAPI). Untreated FAK-WT or FAK −/− cells were used as a negative (15 puncta per cell) and positive (58 puncta per cell) control respectively and the number of Src positive puncta per cell was calculated after treatment with each inhibitor. Each image taken by the Scan-R was also examined to identify any general phenotypic changes, cell death or focusing issues. Of the 80 inhibitors tested 20 induced an obvious change in the localization of active Src and/or a significant reduction in the number of puncta detected. These inhibitors were then validated as described below.

### 2.5 Immunofluorescence

Cells were plated on glass coverslips overnight, treated with inhibitor for 24 h then fixed and stained as described above. Inhibitors were used at the following concentrations: 3MA (10 mM), dasatinib (100 nM), SB218078 (A; 1 μM), PD407824 (B; 10 μM), SB216763 (C; 10 μM), BIO (D; 10 μM), ZM306416 (E; 20 μM), Ki8751 (F; 10 μM) and PF4708671 (10 μM). Primary antibodies were diluted 1:100 except paxillin and p-Src Y416, which were used at 1:500. Imaging was carried out using an FV1000 Olympus Confocal Microscope. Image analysis was carried out using ImageJ. Images shown are representative of three separate experiments.

### 2.6 Immunoblotting and immunoprecipitation

Cells were washed with PBS, lysed in either NP40 lysis buffer (50 mM Tris–HCL at pH 8, 150 mM NaCl, 0.5% NP40) or MD Anderson buffer (1% Triton X 100, 50 mM Hepes pH 7.4, 150 mM NaCl, 1.5 mM MgCl_2_ and 1 mM EGTA) containing a protease and a phosphatase inhibitor tablet (Sigma) and centrifuged for 15 min at high speed (16,000 ×*g* at 4 °C min). A protein assay was then carried out using a Micro BCA Protein Assay Kit. 1 mg of lysate was used to collect immune complexes by immunoprecipitation using 2 μg of antibody or control IgG. Beads were washed three times in lysis buffer before a final wash using 0.6 M LiCl was performed. These samples or 20 μg lysate were then supplemented with sample buffer (Tris at pH 6.8, 20% glycerol, 5% SDS, β-mercaptoethanol and bromophenol blue), separated by SDS-PAGE, transferred to nitrocellulose membrane and then immunoblotted with specific antibodies. Blots shown are representative of three experiments.

### 2.7 siRNA-mediated protein knockdown

80 nM Scrambled (Cat no.: D-001206-13-20), Rps6kb1 1 or Rps6kb1 2 siRNA (Cat no.: D-040893-02 and D-040893-04) were transiently transfected into FAK −/− cells using HiPerFect. Cells were left for 24 h then re-plated onto glass coverslips or 100 mm tissue culture plates for a further 24 h prior to immunofluorescence or immunoblotting.

### 2.8 Statistical tests

For all experiments shown n = 3. Quantification was carried out by counting 50 cells and calculating the percentage of cells with each phenotype after each experiment. Graphs shown throughout the paper show the average percentage and error bars all represent s.d. A Student's T-Test was performed where appropriate to calculate the statistical significance and p values are stated in the Figure Legends.

## 3 Results

### 3.1 p70S6K is regulated by FAK and is involved in Src's spatial regulation

**Fig. 1:**
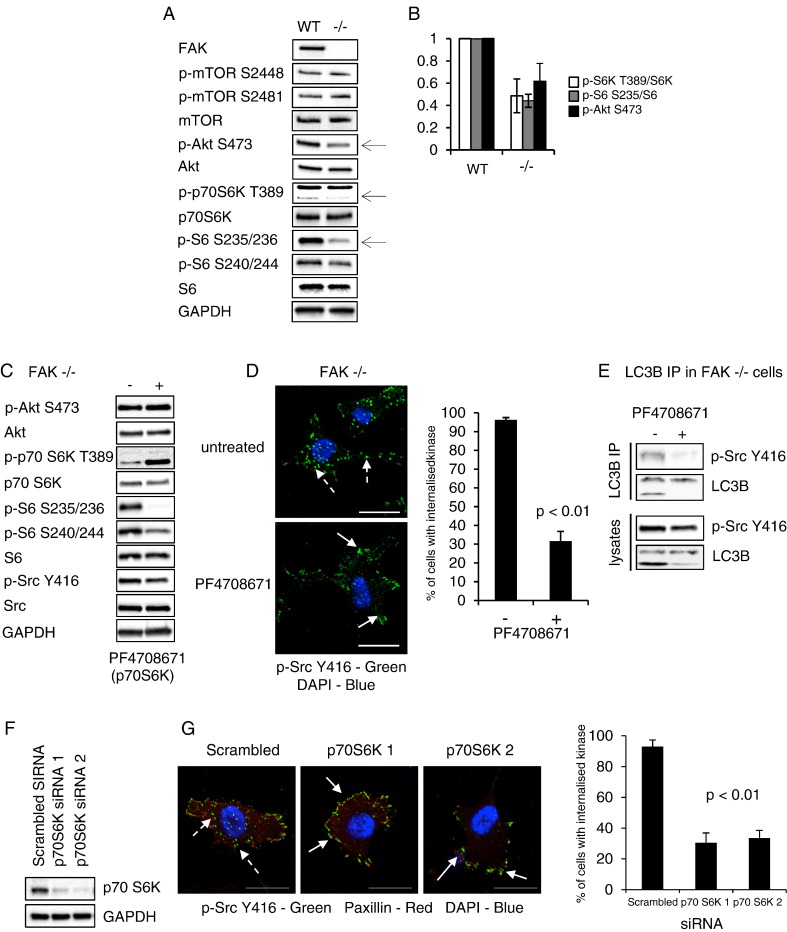
Src trafficking to autophagosomes is dependent upon p70S6K. (A) Cell lysates from SCC FAK-WT and FAK −/− cells were immunoblotted with anti-p-mTOR S2448, anti-p-mTOR S2481, anti-mTOR, anti-p-Akt S473, anti-Akt, anti-p-p70S6K T389, anti-p70S6K, anti-p-S6 S235/236, anti-p-S6 S240/244, anti-S6 and anti-GAPDH. (B) Graph shows the relative ratio of p-p70S6K/total p70S6K, p-S6 S235/236/total S6 and p-Akt S473/total Akt in FAK-WT and FAK −/− cells. Results are presented as mean ± s.d. and significance is p < 0.01 (n = 8). (C) FAK −/− cells were treated with the p70S6K inhibitor PF4708671 (10 μM) for 24 h. Cell lysates were immunoblotted with anti-p-Akt S473, anti-Akt, anti-p-p70S6K T389, anti-p70S6K, anti-p-S6 S235/236, anti-p-S6 S240/244, anti-S6, anti-p-Src Y416, anti-Src and anti-GAPDH. (D) Cells were also fixed and stained for anti-p-Src Y416 (green) and DAPI (blue). Broken arrows show active Src localising to puncta while solid arrows indicate active Src localising to adhesions. Quantification shows the percentage of cells with active Src localising to intracellular puncta. Results are presented as mean ± s.d. and significance is p < 0.01 (n = 3). (E) LC3B was immunoprecipitated from FAK −/− cells treated with PF4708671 and immunoblotting performed with anti-p-Src Y416 and anti-LC3B. (F) FAK −/− cells were transfected with either 80 nM Scrambled, p-70 S6K 1 or p-70 S6K 2 siRNA for 48 h. Immunoblotting carried out using anti-p70S6K and anti-GAPDH antibodies. (G) Cells were also fixed and stained for anti-p-Src Y416 (green), anti-paxillin (red) and DAPI (blue). Broken arrows show active Src localising to puncta while solid arrows indicate active Src localising to adhesions. Scale bars: 20 μm. Quantification shows the percentage of cells with active Src localising to intracellular puncta. Results are presented as mean ± s.d. and significance is p < 0.01 (n = 3).

In examining effects of FAK depletion upon signalling pathways, we found that phosphorylation of Akt (S473), p70S6K (T389) and its downstream target S6 (S235/236 and S240/244) were all reduced in FAK −/− SCCs, while phosphorylation of mTOR (S2448; a key determinant of mTOR activation [12] or its auto-phosphorylation site (2481)) were unaffected (Fig. 1A; reductions quantified in Fig. 1B). This shows that signalling to Akt, p70S6K and S6 was at least partly dependent on FAK in SCC cells.

In previous work, we showed that p-Src predominantly localised to distinctive cytoplasmic puncta in FAK-deficient SCC cells (Supplementary Fig. 1A, broken arrows, quantified in right panel), and this was rescued by re-expression of wild type FAK (FAK-WT), causing p-Src to be restored at focal adhesions (Supplementary Fig. 1A, solid arrows, quantified in right panel). The cytoplasmic punctate structures that are prominent in FAK-deficient cells also contain autophagy proteins such as LC3B and ATG proteins, but not paxillin [5,10]. Since p70S6K has previously been implicated in the regulation of specific forms of autophagy [13–16], we addressed whether the trafficking of active Src to autophagic puncta in FAK-deficient SCC cells was dependent on p70S6K by first using the pharmacological inhibitor PF4708671. As expected, this caused decreased p-S6 (S235/236 and S240/244; Fig. 1C), despite increased levels of p-p70S6K (perhaps by inhibition of a negative feedback mechanism) and impaired sequestration of p-Src into autophagosomes; instead, p-Src was restored at focal adhesions (Fig. 1D, solid arrows, quantified in right panel). Importantly, the p70S6K inhibitor did not affect p-Src levels in FAK −/− cells as judged by immunoblotting (Fig. 1E, lower panels). We also previously showed that LC3B is present in a complex with p-Src in the absence of FAK [5] (Supplementary Fig. 1D), providing biochemical evidence of the formation of an autophagy-associated, Src-containing complex. The LC3B/p-Src Y416 complex was substantially reduced in FAK-deficient cells upon p70S6K inhibitor treatment (Fig. 1E), providing biochemical evidence of impaired Src-selective autophagy. Since kinase inhibitors are rarely specific, we efficiently depleted p70S6K in FAK-deficient cells using two specific siRNAs (Fig. 1F). Loss of p70S6K protein resulted in significant reduction in the number of p-Src-containing puncta and restoration of active Src to paxillin-containing focal adhesions (Fig. 1G, broken arrows, quantified in right panel). Taken together, these data indicate that p70S6K controls Src's autophagic targeting when FAK is absent.

### 3.2 Identifying pharmacological agents that inhibited Src's autophagic targeting

**Fig. 2:**
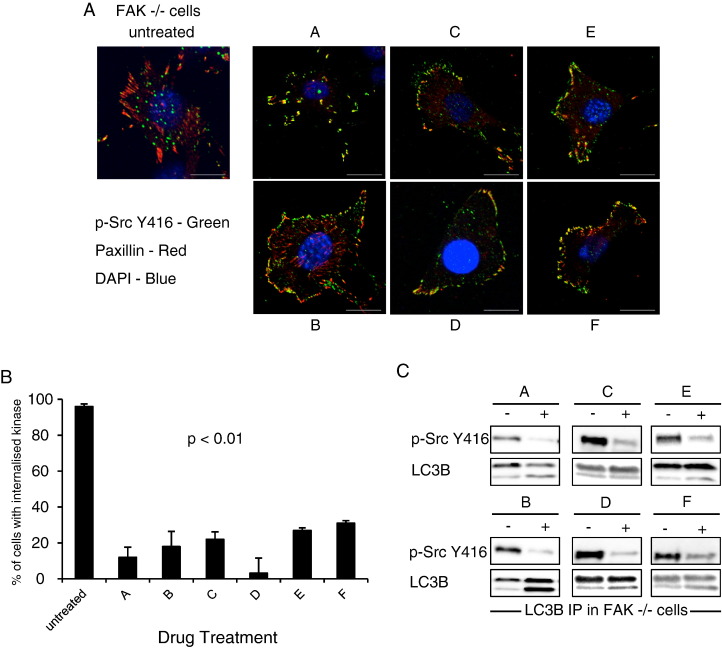
Kinase inhibitors of Src-selective autophagy. (A) FAK −/− cells were treated with the inhibitors A–F for 24 h. Cells were fixed and stained with anti-p-Src Y416 (green), anti-paxillin (red) and DAPI (blue). Scale bars: 20 μm. (B) Percentage of cells with active Src localising to intracellular structures was quantified and data presented as mean ± s.d. Significance for all treatments is p < 0.01 (n = 3). (C) LC3B was immunoprecipitated from FAK −/− cell lysates treated with kinase inhibitors. Then immunoblotting was performed using anti-p-Src Y416 and anti-LC3B antibodies.

Next we performed a small drug screen using a commercially available kinase inhibitor library. Adherent FAK −/− cells were treated with 80 different kinase inhibitors (Tocriscreen Kinase Inhibitor Toolbox) for 24 h, then fixed and stained with p-Src Y416 antibody, Deep Red Cell Mask and DAPI, so as to visualise autophagosomes and adhesions, cell cytoplasm and nucleus, respectively. Cells were imaged and analysed using an Olympus Scan-R microscope to calculate the number of Src-positive puncta per cell in untreated FAK-WT (less than 15 puncta per cell) and untreated FAK −/− (58 puncta per cell) cells as negative and positive controls. We focused on 6 chemical entities that significantly reduced the number of Src-positive puncta detected per cell (Fig. 2A and quantified in 2B), but were not in the class of chemicals that would inhibit Src phosphorylation per se (for example, there was no significant decrease in either the amount of total or active Src after treatment of FAK-WT cells with these agents) (Supplementary Fig. 1C, middle and upper panels respectively). Moreover, the formation of the LC3B/Src complex was reduced after treatment with all 6 kinase inhibitors, providing biochemical evidence of inhibition of Src's targeting to intracellular puncta that supported the imaging data (Fig. 2C and lysates shown in Supplementary Fig. 1C and 1E). The screen was validated using inhibitors that are known to block general autophagy (3MA [17]) or Src phosphorylation (dasatinib [5]), and these, as expected, blocked visualisation of active Src at autophagic puncta in FAK −/− cells (Supplementary Fig. 1B). For the 6 inhibitors we identified, termed A–F, we wished to take an unbiased view on their mode of action since we believed that they are relatively non-specific kinase inhibitors (details of these inhibitors can be found in Table 1). Next, we examined how these affected the Akt/p70S6K/S6 pathway (and PDK1 upstream) that we knew to control Src's autophagic targeting (Fig. 1).

### 3.3 Kinase inhibitors selected to disrupt Src-selective autophagy inhibit signalling to PDK1, Akt, p70S6K and S6

**Fig. 3:**
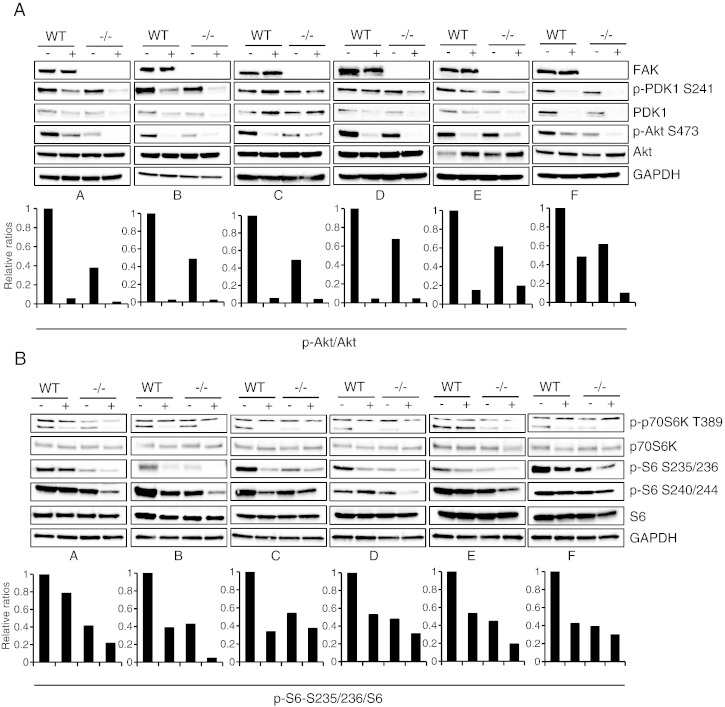
The PDK1/Akt/p70S6K pathway is linked to Src-selective autophagy. FAK-WT and FAK −/− cells were treated with the inhibitors A–F for 24 h. (A) Cell lysates were immunoblotted with anti-FAK, anti-p-PDK1 S241, anti-PDK1, anti-p-Akt S473, anti-Akt and anti-GAPDH. Graphs show the relative ratio of phospho-Akt S473/total Akt. (B) Cell lysates were immunoblotted with anti-p-p70S6K T389, anti-p70S6K, anti-p-S6 S235/236, anti-p-S6 S240/244, anti-S6 and anti-GAPDH. Graphs show the relative ratio of p-S6 S235/236/total S6.

We investigated the effects of the 6 pharmacological agents (A–F). All inhibitors (with the exception of one, namely inhibitor C) caused a robust reduction in p-PDK1 and total PDK1, particularly notable in FAK-deficient cells. Treatment with inhibitor C resulted in a small increase in total PDK1 in FAK −/− cells (Fig. 3A). All 6 inhibitors caused reduced phosphorylation of Akt, most prominently in FAK-deficient cells (Fig. 3A, quantified in lower panels).

Akt signalling is reported to suppress autophagy in response to mitogens, via activation of mTOR [18]. In this latter study, PDK1 phosphorylated and activated Akt, which in turn phosphorylated and inhibited the activity of TSC1/TSC2 complex — a negative regulator of mTOR [18]. Since the PDK1/Akt pathway has been shown to influence autophagy via mTOR in multiple contexts, we used phospho-specific antibodies to study the expression and phosphorylation of mTOR at Ser2448, a key determinant of its activity [12], and at its autophosphorylation site Ser2481. We found that both mTOR expression and phosphorylation were unaffected by any of the 6 kinase inhibitors (Supplementary Fig. 2), demonstrating that steady state mTOR activity was not influenced by agents that inhibit Src's autophagic targeting. We then investigated the effect of the 6 inhibitors on phosphorylation of p70S6K and on the phosphorylation of its S6 substrate. All of the inhibitors showed remarkable consistency in inhibiting the phosphorylation of: a) p70S6K (Fig. 3B, faster migrating species), and b) S6K on S235/236 and S240/244. Residual phosphorylation was barely detectable by immunoblotting in the FAK −/− cells (Fig. 3B, quantification is shown for the ratio of p-S6 S235/236/S6 in lower panels).

### 3.4 Autophagosomes act as sub-locale for signalling to p70S6K

**Fig. 4:**
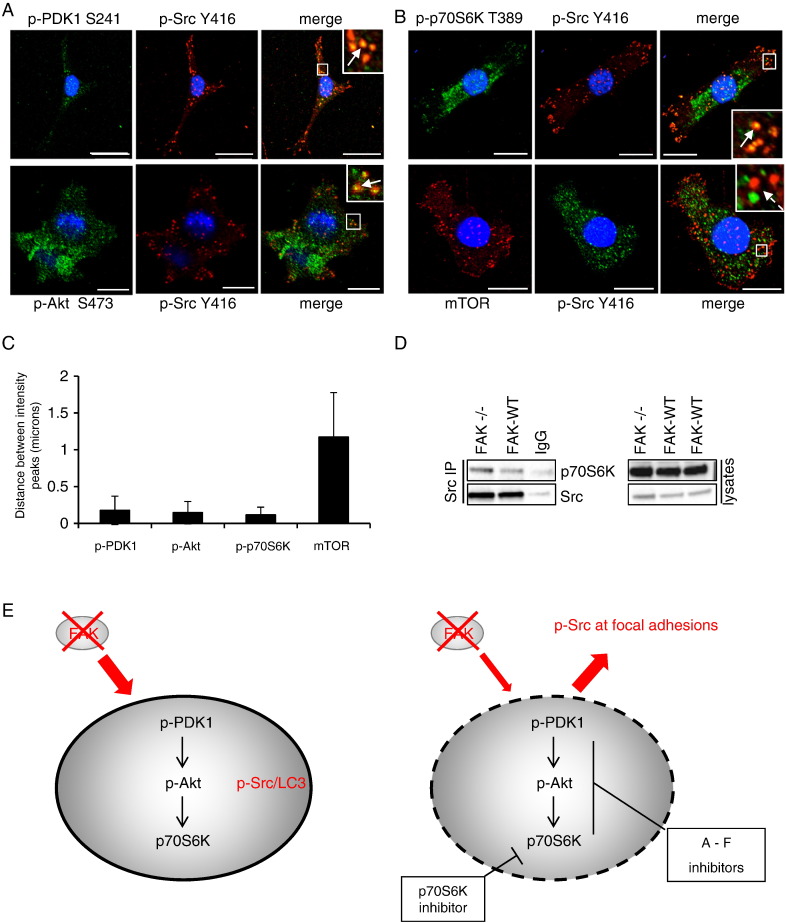
Signalling proteins localise to Src-containing autophagosomes. FAK −/− cells were fixed and stained with (A) anti-p-PDK1 S241 (upper panels), anti-p-Akt S473 (lower panels) or (B) anti-p-p70S6K T389 (upper panels), anti-mTOR (lower panels) and with anti-p-Src Y416 and DAPI (blue). Merged and zoomed images are shown. Solid arrows indicate co-localisation while broken arrows show its absence. Scale bars: 20 μm. (C) Amount of co-localisation per cell was calculated by measuring by the distance between the intensity peaks of the different fluorescent signals. Results are presented as mean ± s.d. (n = 20 intensity peaks from 5 cells). (D) Src was immunoprecipitated from SCC cells and immunoblotting performed with anti-p70S6K and anti-Src. (E) Model of signalling at Src positive autophagosomes. Schematic depicting how the use of inhibitors influences the trafficking of active Src to autophagic puncta in the absence of FAK.

We next examined the intracellular localisation of activated components of the PDK1/Akt/p70S6K signalling pathway. We found that all of these co-localised to autophagic puncta with active Src (Fig. 4A and B respectively, solid arrows in merged images). Although we observed mTOR in cytoplasmic puncta, it did not visibly co-localise with p-Src-containing puncta in FAK-deficient cells (Fig. 2B, lower panels, broken arrow in merged image). A measure of the co-localisation (or lack thereof in the case of mTOR) is presented as distance between intensity peaks of co-stained proteins (Fig. 2C). We also identified a biochemical complex that contained both Src and p70S6K, although we have no evidence that this complex is via direct binding (Fig. 4D). These data imply that the intracellular puncta that contain autophagy proteins also physically serve as a sub-locale for the activated kinase components of the signalling pathway – PDK1/Akt/p70S6K – which promotes efficient targeting of Src to these structures when Src cannot be tethered at focal adhesion complexes in the absence of FAK.

Taken together, our results imply that the trafficking of active Src to autophagosomes in FAK-deficient cancer cells is controlled by the PDK1/Akt/p70S6K pathway. Chemical agents that inhibit Src-selective autophagy (Fig. 2) also inhibit signalling to PDK1/Akt/p70S6K/S6, and FAK −/− cells are considerably more sensitive to inhibition (Fig. 3). Moreover, inhibiting p70S6K results in a block to Src's autophagic targeting (Fig. 1). Finally, the phosphorylated components of the PDK1/Akt/p70S6K pathway co-exist with active Src at autophagic puncta that are dependent on p70S6K (Fig. 4; model depicted in Fig. 4E).

## 4 Discussion

In this study, we set out to determine the signalling determinants of the trafficking of active Src to autophagosomes in FAK-depleted SCC cancer cells. We identified 6 kinase inhibitors that efficiently blocked the trafficking of active Src to autophagosomes, and all 6 inhibited signalling to PDK1/Akt/p70S6K/S6, without affecting mTOR phosphorylation. The basal levels of p-p70S6K, p-S6K and p-Akt were lower in untreated FAK-deficient cells, providing the first evidence that FAK is required for optimal signalling to p70S6K; we found that FAK-deficient cells were more sensitive to p70S6K inhibition.

By using a pharmacological inhibitor and siRNA targeting p70S6K, we confirmed that p70S6K is required for the targeting of active Src to autophagosomes in the absence of FAK, whereas p70S6K-mediated phosphorylation of S6 has previously been correlated with autophagy suppression [13]. However, there are also multiple examples of p70S6K-mediated promotion of autophagy. These include studies in *Drosophila* where dS6K is required for starvation-induced autophagy [14], in mammalian cells where resveratrol-induced inhibition of p70S6K impairs the autophagic response [15], and in colorectal cancer cells where p70S6K positively regulates 6-thioguanine-induced autophagy [16]. Src's autophagic targeting under ‘adhesion stress’ upon deletion of FAK is therefore another example of dependence on signalling pathways that converge on p70S6K.

The regulation of Src's localization to autophagosomes by the PDK1/Akt/p70S6K pathway is accompanied by the co-localisation of components of this signalling pathway to Src-containing autophagosomes, but not mTOR. This implies that the phosphorylation events required for the recruitment of Src occur at these structures, or during their assembly or formation. We therefore propose these autophagosomes represent sub-cellular locale that house the signalling ‘module’ that activates p70S6K in a spatially-regulated manner, and, in turn, this is required for efficient targeting of Src to these structures when FAK is absent (schematic shown in Fig. 4E). This is another example of signalling components being found at autophagosomes; a recent study reported how ATG5/12-positive pre-autophagosomes and LC3B-positive membranes can act as cellular signalling hubs that facilitate the spatial co-ordination and activation of the MEK/ERK cascade in NIH 3T3 cells [19].

## 5 Conclusions

We have established that: a) p-Akt, p-p70S6K and p-S6K are reduced in untreated FAK-deficient SCC cells, and b) inhibition of the PDK1/Akt pathway leads to inactivation of p70S6K, reduced p-S6K downstream, and restoration of active Src to focal adhesions in the absence of FAK (depicted in model Fig. 4). Unlike classical autophagy signalling, this process did not involve changes to mTOR phosphorylation and it may be that components of these pathways are able to activate p70S6K directly, as PDK1 has been shown to directly phosphorylate and activate p70S6K in vitro and in vivo [20]. We conclude that p70S6K is required for the sequestration of active Src away from focal adhesions and into autophagic puncta, since knock down of its expression and a specific kinase inhibitor induces re-localization of Src to peripheral adhesions. We also conclude that signalling to p70S6K in SCC cancer cells is FAK-dependent, and that inhibition of p70S6K activation by drugs that target the upstream kinase pathways is more efficient when FAK is absent.

## Conflict of interest

The authors declare no conflict of interest.

## Figures and Tables

**Table 1:** A–F inhibitors in this study from Tocriscreen Kinase Inhibitor Toolbox. SCC cells were plated overnight then the inhibitors from the Tocriscreen Kinase Inhibitor Toolbox were added at 1 μM, 10 μM or 20 μM for 24 h. Plates were fixed and stained with anti-p-Src Y416, Deep Red Cell Mask and DAPI, then imaged using a Scan-R microscope. Scan-R analysis software was used to calculate the number of puncta (based on size and shape of Src positive puncta) per cell (calculated using DAPI) and to identify any alterations to the intracellular localisation of active Src. The effect that each inhibitor had on active Src is summarised in this table.

Inhibitor	Inhibitor name	Target	20 μM	10 μM	1 μM
A	SB218078	Chk1	Less p-Src Y416 puncta	Less p-Src Y416 puncta	Less p-Src Y416 puncta
B	PD407824	Chk1	Toxic for cells	Cells visibly unhealthy	Less p-Src Y416 puncta
C	SB216763	GSK-3β	Less p-Src Y416 puncta	Less p-Src Y416 puncta	No detectable change
D	BIO	GSK-3β	Toxic for cells	Less p-Src Y416 puncta	Less p-Src Y416 puncta
E	ZM306416	VEGFR	Less p-Src Y416 puncta	No detectable change	No detectable change
F	Ki8751	VEGFR	Less p-Src Y416 puncta	Less p-Src Y416 puncta	Less p-Src Y416 puncta

Note: The above named inhibitors, designated A–F, were used as agents to inhibit Src-selective autophagy in the absence of FAK. We think it is likely that these may work in this context by off target effects.

## Supplementary data

Supplementary Fig. 1: Src phosphorylation is not reduced by kinase inhibitors A–F. (A) FAK −/− and FAK-WT SCC cells were fixed and stained with anti-p-Src Y416 antibody (green) and DAPI (blue). Broken arrows show active Src in puncta while solid arrows indicate active Src in focal adhesions. Quantifications show percentage of cells with internalised Src kinase in puncta. Results are presented as mean ± s.d. and significance in p < 0.001 (n = 3). (B) FAK −/− cells were treated with 3MA (10 mM for 48 h) or dasatinib (100 nM for 24 h). Cells were fixed and stained with anti-p-Src Y416 (green), anti-paxillin (red in left panels) and DAPI (blue). Broken arrows show active Src in puncta while solid arrows indicate active Src in focal adhesions. (C) FAK-WT and FAK −/− cells were treated with various kinase inhibitors A–F and lysates immunoblotted using anti-p-Src Y416, anti-Src and anti-GAPDH antibodies. (D) LC3B was immunoprecipitated from FAK −/− cells then immunoblotted with anti-p-Src Y416 and anti-LC3B antibodies. IgG control is shown. (E) FAK −/− cells were treated with various kinase inhibitors A–F and lysates immunoblotted using anti-LC3B antibodies. Supplementary Fig. 2: Kinase inhibitors A–F do not affect mTOR phosphorylation. FAK-WT and FAK −/− cells were treated with various kinase inhibitors A–F and lysates immunoblotted using anti-p-mTOR S2448, anti-p-mTOR S2481, anti-mTOR and anti-GAPDH antibodies.
