# Supplementary material for: p70S6K is regulated by focal adhesion kinase and is required for Src-selective autophagy
Source: Cell Signal. 2015 Sep;27(9):1816–23. doi: 10.1016/j.cellsig.2015.05.017 (PMC4508348; doi:10.1016/j.cellsig.2015.05.017)

Supplementary Figure 1 Src phosphorylation is not reduced by kinase inhibitors A-F

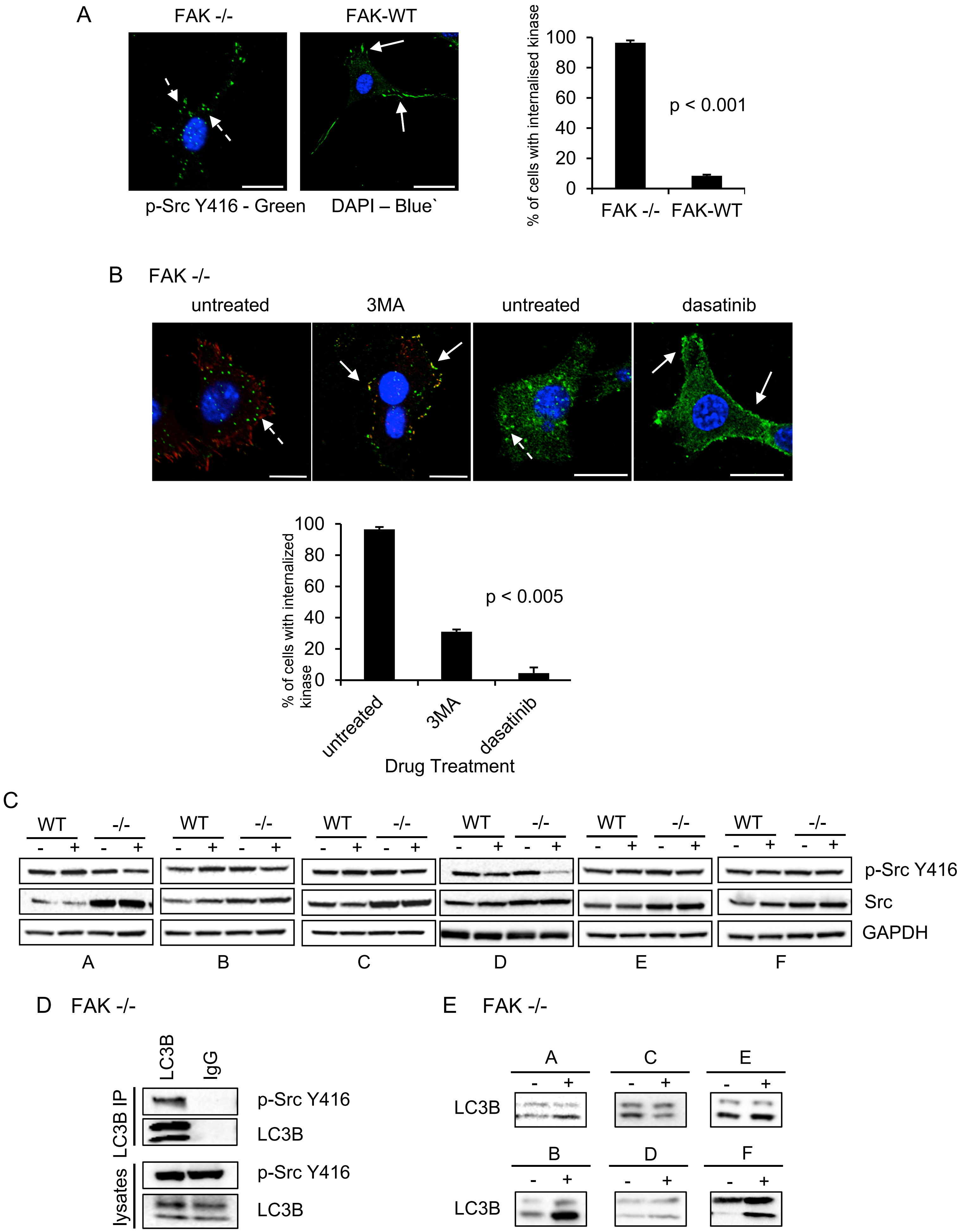

Supplementary Figure 2   Kinase inhibitors A-F do not affect mTOR phosphorylation

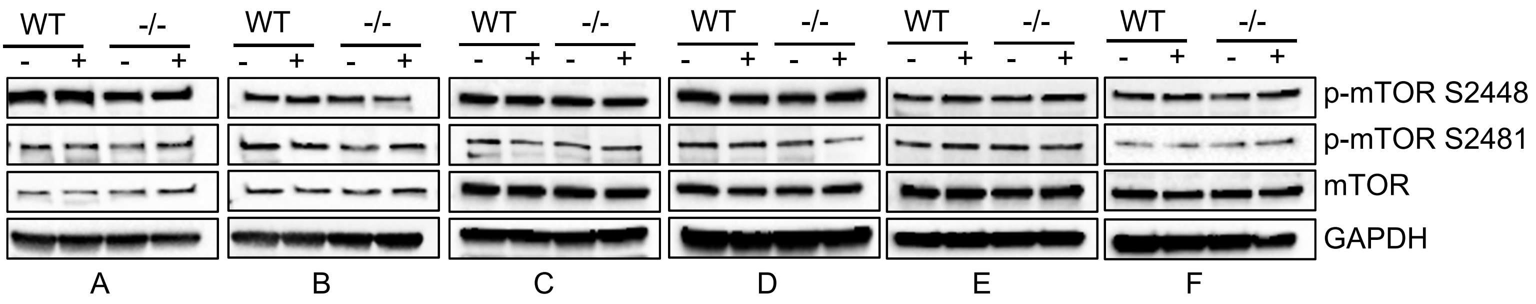

Supplement: Supplementary file 1 — Supplementary Fig. 1: Src phosphorylation is not reduced by kinase inhibitors A–F. (A) FAK −/− and FAK-WT SCC cells were fixed and stained with anti-p-Src Y416 antibody (green) and DAPI (blue). Broken arrows show active Src in puncta while solid arrows indicate active Src in focal adhesions. Quantifications show percentage of cells with internalised Src kinase in puncta. Results are presented as mean ± s.d. and significance in p < 0.001 (n = 3). (B) FAK −/− cells were treated with 3MA (10 mM for 48 h) or dasatinib (100 nM for 24 h). Cells were fixed and stained with anti-p-Src Y416 (green), anti-paxillin (red in left panels) and DAPI (blue). Broken arrows show active Src in puncta while solid arrows indicate active Src in focal adhesions. (C) FAK-WT and FAK −/− cells were treated with various kinase inhibitors A–F and lysates immunoblotted using anti-p-Src Y416, anti-Src and anti-GAPDH antibodies. (D) LC3B was immunoprecipitated from FAK −/− cells then immunoblotted with anti-p-Src Y416 and anti-LC3B antibodies. IgG control is shown. (E) FAK −/− cells were treated with various kinase inhibitors A–F and lysates immunoblotted using anti-LC3B antibodies. Supplementary Fig. 2: Kinase inhibitors A–F do not affect mTOR phosphorylation. FAK-WT and FAK −/− cells were treated with various kinase inhibitors A–F and lysates immunoblotted using anti-p-mTOR S2448, anti-p-mTOR S2481, anti-mTOR and anti-GAPDH antibodies. [file mmc1.pdf]
